# A Portable Gluten Sensor for Celiac Disease Patients May Not Always Be Reliable Depending on the Food and the User

**DOI:** 10.3389/fnut.2021.712992

**Published:** 2021-07-20

**Authors:** Alena Marić, Katharina Anne Scherf

**Affiliations:** Department of Bioactive and Functional Food Chemistry, Institute of Applied Biosciences, Karlsruhe Institute of Technology, Karlsruhe, Germany

**Keywords:** barley, celiac disease, enzyme-linked immunosorbent assay, gluten-free, rye, sensor, wheat

## Abstract

A strict lifelong gluten-free (GF) diet is currently the only known effective treatment for celiac disease (CD), an inflammatory disorder of the small intestine with a worldwide prevalence of about 1%. CD patients need to avoid wheat, rye, and barley and consume GF foods containing <20 mg/kg of gluten. However, strict adherence to a GF diet tends to reduce the quality of life of CD patients compared to the general population and may lead to fear of inadvertent gluten consumption, especially when eating out. To help alleviate risk of gluten exposure, a portable gluten sensor was developed by Nima Labs that allows CD patients to test foods on site prior to consumption. With very limited independent information on the analytical performance of the Nima sensor available so far, our aim was to evaluate the reliability of the sensor using a variety of different foods with defined gluten content. All samples were tested with the sensor and analyzed by enzyme-linked immunosorbent assay as reference method. Of the 119 samples with gluten content ranging from 2 to 101,888 mg/kg tested in total, the sensor showed 80 positive (67.2%), 37 negative (31.1%) and 2 invalid results at the first of three consecutive measurements. The detection rate for samples containing ≥20 mg/kg of gluten was 90%. Samples containing 2 mg/kg of gluten or below consistently tested negative, but samples with a gluten content between 2 to 20 mg/kg of gluten may either test positive or negative. Overall, the performance of the sensor was acceptable in our study, but we observed systematic variation between different users that also appeared to depend on the sample being tested. This highlights the need to improve user education especially regarding the effect of sampling, testing limitations in case of partially hydrolyzed, fractionated or fermented gluten and training users on how to perform the test in a way that gluten will be reliably detected.

## Introduction

Celiac disease (CD) is one of the most common food-induced inflammatory diseases affecting about 1% of the population worldwide (1). It is triggered in genetically susceptible individuals by the storage proteins of wheat, rye, and barley, which are referred to as gluten. The ingestion of gluten-containing cereals leads to small intestinal inflammation with villous atrophy, infiltration of intraepithelial lymphocytes and subsequently a variety of different intra- and extraintestinal symptoms (2). With a strict lifelong gluten-free (GF) diet as the only effective treatment available so far, CD patients need to avoid products made of wheat, rye, barley and closely related crosses or varieties. Next to naturally GF foods, CD patients may consume specific products bearing a GF label according to national legislation. As laid down in Codex Alimentarius Standard 118-1979, foods labeled “gluten-free” may contain no more than 20 mg of gluten per kg of the product (3). To ensure compliance with the limit, it is essential that GF cereals and pseudo cereals are not mixed with gluten-containing cereals from cultivation to processing into the final product, that GF dishes are prepared separately from gluten-containing dishes in large kitchens and restaurants, and that the methods for gluten analysis are reliably applied by manufacturers of GF products and food control authorities (4).

However, strict adherence to a GF diet is associated with significant restrictions for those affected, which lead to a reduced quality of life compared to the general population (5) and can even provoke anxiety and depression (6). Social activities, eating out and traveling are perceived as particularly problematic, especially in the first years after diagnosis. On these occasions, up to 88% of respondents deliberately accepted dietary transgressions because the GF diet is perceived as too strict, difficult and uncomfortable during social activities (7). Thus, CD patients risk a recurrence of symptoms and consequently an increased risk of long-term complications (8).

So far, methods for analyzing gluten traces in food are designed exclusively for use in specialized laboratories. Because CD patients cannot always be sure that GF foods are really GF, especially when eating out, there is a need for point-of-care (POC) tests. Ideally, small and portable POC tests should provide low-cost, fast, and accurate results with small sample volumes and be easy to perform so that consumers can use them without problems, e.g., in a restaurant. Often, such POC tests are connected to a smartphone app and a social media presence (9).

The POC test for gluten detection used in this study has recently been developed for CD patients by Nima Labs (10). The sensor performs sample preparation, gluten analysis, result interpretation and data transmission within 2–4 min and displays a wheat ear (positive, gluten detected) or smiley (negative, no gluten detected) symbol. Positive was defined by the manufacturer as the sensor detecting 20 mg/kg of gluten or more in sample amounts of 0.1–2 g with a 99.0% probability as true positive. In contrast, negative was defined as <2 mg/kg gluten. Thus, there is a measurement uncertainty in the range of 2–20 mg/kg gluten. The sensor is based on two monoclonal antibodies 13F6 and 14G11 directed against the 33-mer peptide from α-gliadin immobilized on the test line of a lateral flow immunoassay (LFIA). Users are instructed to place a pea-sized portion of the food into a disposable capsule and activate the grinding mechanism when screwing the top of the capsule shut to homogenize the test portion. The extraction solution is added with the last turn. After inserting the capsule into the instrument and pressing the start button, the test portion is mixed with the extraction solution for 30 s and a valve finally allows the extract to flow onto the LFIA. A peak identification algorithm compares the differences in light intensities of the negative LFIA with those of the test line of a positive LFIA, taking into account a control line and a hook line (at very high gluten concentrations).

Various factors such as extraction time, sensitivity and specificity of the two antibodies, cross-reactivities, reproducibility, food matrix, sample weight and sample inhomogeneity were taken into account by the manufacturer. The analysis of 447 food samples gave three false negative results, ten false positive results and 31 invalid results, which occur when a test is not completed correctly, e.g., because the food to be tested absorbs the entire volume of extraction solution, the solution becomes too viscous or the pores of the LFIA become blocked (10). Independent tests with the sensor on 13 different products showed that in 96.5% of the tests the samples with 20 mg/kg of gluten or more were identified as true positives. In some samples, such as bread, pasta and puffed maize, only 47% of the samples with 20 mg/kg of gluten were identified as true positive and the detection rate increased to 88% at 30 mg/kg of gluten and to 97.5% at 40 mg/kg of gluten (11).

Further studies to assess the reliability of the sensor are not yet available, but are essential as false positives restrict CD patients' options to compose their meal and have a negative impact on the GF food industry, while false negatives pose a significant risk to CD patients (12). Points that have not been studied so far include the possibility of a hook effect occurring at high gluten concentrations and the problem of sampling in the case of inhomogeneous distribution of gluten in food and dishes. The sensor was designed to detect intact gluten proteins and there have been no studies to date on whether it also detects fermented or partially hydrolyzed gluten. Since the study by Taylor et al. (11) used wheat flour only to produce defined food samples, there is also a lack of knowledge about the sensitivity and specificity of the sensor to rye and barley.

The main aim of our study was therefore to test the reliability of the portable gluten sensor using homogeneous and inhomogeneous samples with defined gluten content. We used naturally GF raw materials and prepared foods from different categories with defined gluten content by blending in different gluten sources (wheat, rye, and barley flours). Commercially available foods (*n* = 21, nine of them bearing a GF label) containing fermented or partially hydrolyzed gluten were also analyzed. A second aim was to study the influence of sample weight, high gluten content and different users on the results of the sensor.

## Methods

### Material

All chemicals, reagents and solvents such as acetonitrile, disodium hydrogen phosphate, dithiothreitol, ethanol, potassium dihydrogen phosphate, 1-propanol, sodium chloride, trifluoroacetic acid (TFA) and urea were at least *pro analysi* or HPLC grade. Cocktail (patented) was from R-Biopharm (Darmstadt, Germany). The Prolamin Working Group (PWG)-gliadin reference material (13) was obtained from the Arbeitsgemeinschaft Getreideforschung e.V. (Association of Cereal Research, Detmold, Germany). Organic grains of wheat and rye were from denree (Töpen, Germany) and those of barley from Davert (Aschberg, Germany). GF rice flour was from Müller's Mühle (Gengenbach, Germany). All other foods and ingredients used to prepare food samples with defined gluten content were purchased in a local supermarket (Karlsruhe, Germany). Commercially available products with unknown gluten concentrations (nine beers B1-B9, four sauces S1-S4, three potato products P1-P3, two tofu T1-T2, and three sourdough samples D1-D3) from different manufacturers were also bought in a local supermarket (Karlsruhe, Germany). Some of these products had a GF label according to European Commission Implementing Regulation (EU) No. 828/2014.

### Determination of Gluten Content in Wheat, Rye, and Barley Flours

Wheat, rye, and barley grains were milled into wholemeal flours using a variable speed rotor mill (Pulverisette 14, Fritsch, Idar-Oberstein, Germany) and a 500 μm sieve. Wheat flour was used without additional sieving. Rye and barley flours were used both without additional sieving and with additional sieving (500 μm) to improve homogeneity of the food samples (designated as rye II and barley II).

After a 2-week rest, the gluten content was determined according to modified Osborne fractionation combined with reversed-phase high-performance liquid chromatography (RP-HPLC) as described by Lexhaller et al. (14). In brief, the flours (100 mg) were extracted sequentially by vortex mixing for 2 min at 22°C and magnetic stirring with salt solution (2 × 1 mL; 0.4 mol/l NaCl with 0.067 mol/L Na_2_HPO_4_/KH_2_PO_4_, pH 7.6) for 10 min at 22°C (albumins/globulins), followed by 60% (v/v) ethanol (3 × 0.5 mL) for 10 min at 22°C (prolamins), and glutelin extraction solution [2 × 1 mL; 50% (v/v) 1-propanol/0.1 mol/L Tris-HCl, pH 7.5 containing 2 mol/L urea and 0.06 mol/L (w/v) dithiothreitol] for 30 min at 60°C under argon (glutelins). The suspensions were centrifuged (3,550 × *g*, 25 min, 22°C), the supernatants combined and made up to 2 mL with the extraction solvent, respectively.

The extracts were filtered (Whatman Spartan 13/0.45 RC, GE Healthcare, Freiburg, Germany) and analyzed by RP-HPLC: instrument, UFPLC Prominence with LabSolutions software (Shimadzu, Duisburg, Germany); column, Acclaim 300 C_18_ (particle size 3 μm, pore size 30 nm, 2.1 × 150 mm, Thermo Fisher Scientific, Braunschweig, Germany); temperature, 60°C; injection volume, 20 μL for albumins/globulins, 10 μL for wheat, 20 μL for rye and 40 μL for barley prolamins; 20 μL for wheat and barley and 40 μL for rye glutelins; elution solvents, TFA (0.1%, v/v) in water (A) and TFA (0.1%, v/v) in acetonitrile (B); linear gradient, 0–0.4 min 0% B, 0.5 min 20% B, 7 min 60% B, 7.1–9.0 min 90% B, 9.1–27 min 0% B for albumins/globulins; 0–0.4 min 5% B, 0.5 min 30% B, 18 min 80% B, 18.1–20.1 min 90% B, 20.2–36 min 5% B for prolamins and glutelins; flow rate, 0.4 mL/min; detection, UV absorbance at 210 nm. PWG-gliadin was used for external calibration and the absorbance areas were used to calculate the protein content of the extracts. Gluten content was the sum of prolamin and glutelin content, respectively. Three independent biological replicates were performed for each flour.

### Preparation of Foods With Defined Gluten Content

Typical recipes and kitchen utensils were used to ensure practical relevance of our study. All naturally GF raw materials were confirmed to be GF by R5 sandwich ELISA prior to use (prolamin content below the limit of quantitation at 2.5 mg/kg). A GF control was prepared for each food using only GF ingredients. Then, a gluten-containing mixture was made by adding a defined amount of wheat, rye, and barley flour, respectively, to the GF control to reach a target gluten content of 1,000 mg/kg (mix_1,000_). The mix_1,000_ was further blended with the GF control to a target gluten content of 100 mg/kg (mix_100_). This mix_100_ was subsequently used to adjust the target gluten content to 4 or 5 mg/kg, 10, 20, and 30 mg/kg for high-protein, high-fat and unheated high-starch foods and to 3, 6, 12, 18, and 30 mg/kg for heated high-starch foods (Table 1).

**Table 1:** Overview of high-protein, high-fat and high-starch foods with defined gluten content.

**Code**	**Sample matrix**	**Gluten source (flour)**	**Intended gluten distribution**	**Target gluten content (mg/kg)**				
**High-protein and high-fat foods**								
A	Sausage meat	Wheat	Inhomogeneous		4	10	20	30
B	Meatball	Wheat	Homogeneous		4	10	20	30
C	Meatball	Wheat	Inhomogeneous		4	10	20	30
D	Meatball	Barley	Homogeneous		4	10	20	30
E	Meatball	Rye	Homogeneous		4	10	20	30
F	Meatball	Wheat/rye/barley	Homogeneous		4	10	20	30
G	Vegetarian patty	Wheat	Inhomogeneous		4	10	20	30
H	Salad dressing	Wheat	Inhomogeneous		5	10	20	30
**Unheated high-starch foods**								
I	Rice flour	Wheat	Homogeneous		5	10	20	30
J	Rice flour	Barley	Homogeneous		5	10	20	30
K	Rice flour	Barley, sieved	Homogeneous		5	10	20	30
L	Rice flour	Rye	Homogeneous		5	10	20	30
M	Rice flour	Rye, sieved	Homogeneous		5	10	20	30
N	Rice flour	Durum wheat	Homogeneous		5	10	20	30
O	Rice flour	Spelt	Homogeneous		5	10	20	30
P	Rice flour	Einkorn	Homogeneous		5	10	20	30
Q	Rice flour	Emmer	Homogeneous		5	10	20	30
**Heated high-starch foods**								
R	Rice bread, crumb	Wheat	Homogeneous	3	6	12	18	30
S	Rice bread, crust	Wheat	Homogeneous	3	6	12	18	30
T	Rice bread, crumb	Barley	Homogeneous	3	6	12	18	30
U	Rice bread, crust	Barley	Homogeneous	3	6	12	18	30
V	Rice bread, crumb	Rye	Homogeneous	3	6	12	18	30
W	Rice bread, crust	Rye	Homogeneous	3	6	12	18	30

#### High-Protein and High-Fat Foods

**Figure 1:**
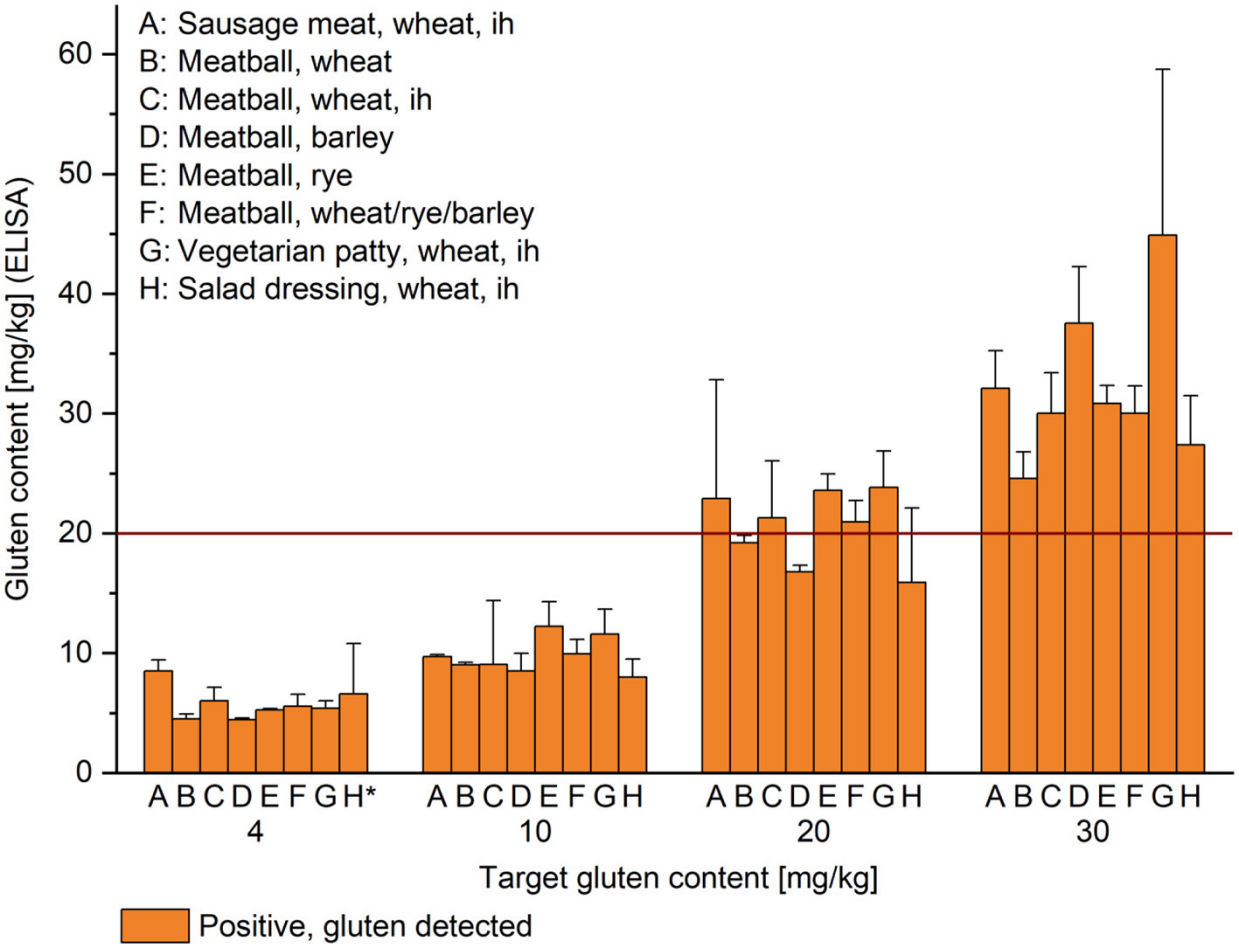
Gluten content of high-protein and high-starch foods. Target gluten content is indicated below the x-axis and gluten content analyzed by R5 sandwich ELISA on the y-axis; given as mean (*n* = 3) + standard deviation. The result of the sensor at the first of three consecutive measurements was gluten detected (orange) in all cases. ih, inhomogeneous, *, target gluten content was 5 mg/kg for sample H. All other samples not designated as inhomogeneous were prepared to be homogeneous. The red horizontal line indicates the threshold for gluten-free products at 20 mg/kg.

Commercially available GF sausage meat (100 g portions) without or with addition of wheat flour was heated in aluminum foil in water at 100°C for 30 min. After cooling to room temperature, the sausage meat was cut and homogenized in an HR 3655/00 blender (Philips, Hamburg, Germany). The final samples made of GF sausage meat and mix_100_ were only blended by hand using mortar and pestle for 30 s with the intent to achieve an inhomogeneous gluten distribution (sample A) (Figure 1).

Meat balls (150 g portions) were prepared from minced meat (50% pork, 50% beef), eggs, chopped onions, salt and without or with addition of wheat, rye or barley flour, respectively, as well as a mix of wheat, rye, and barley flour (1 + 1 + 1, w/w/w). The portions were fried for 7 min on each side in sunflower oil. After cooling, the meat balls were homogenized as described above. Homogeneous samples were blended using mortar and pestle for 3 min (samples B–F).

The vegetarian patty contained soy granules soaked in water, GF rice flour, eggs, chopped onions and salt without or with addition of wheat flour. The mass was divided into 150 g portions and further processed as described for the meat balls, with the exception that final blending only lasted for 30 s (sample G).

The salad dressing contained sunflower oil, vinegar, herbs, salt and sugar without or with addition of wheat flour mixed in the blender. Guar gum was slowly added to achieve high viscosity and the salad dressing was further mixed for 30 s with a spatula (sample H).

#### Unheated High-Starch Foods

**Figure 2:**
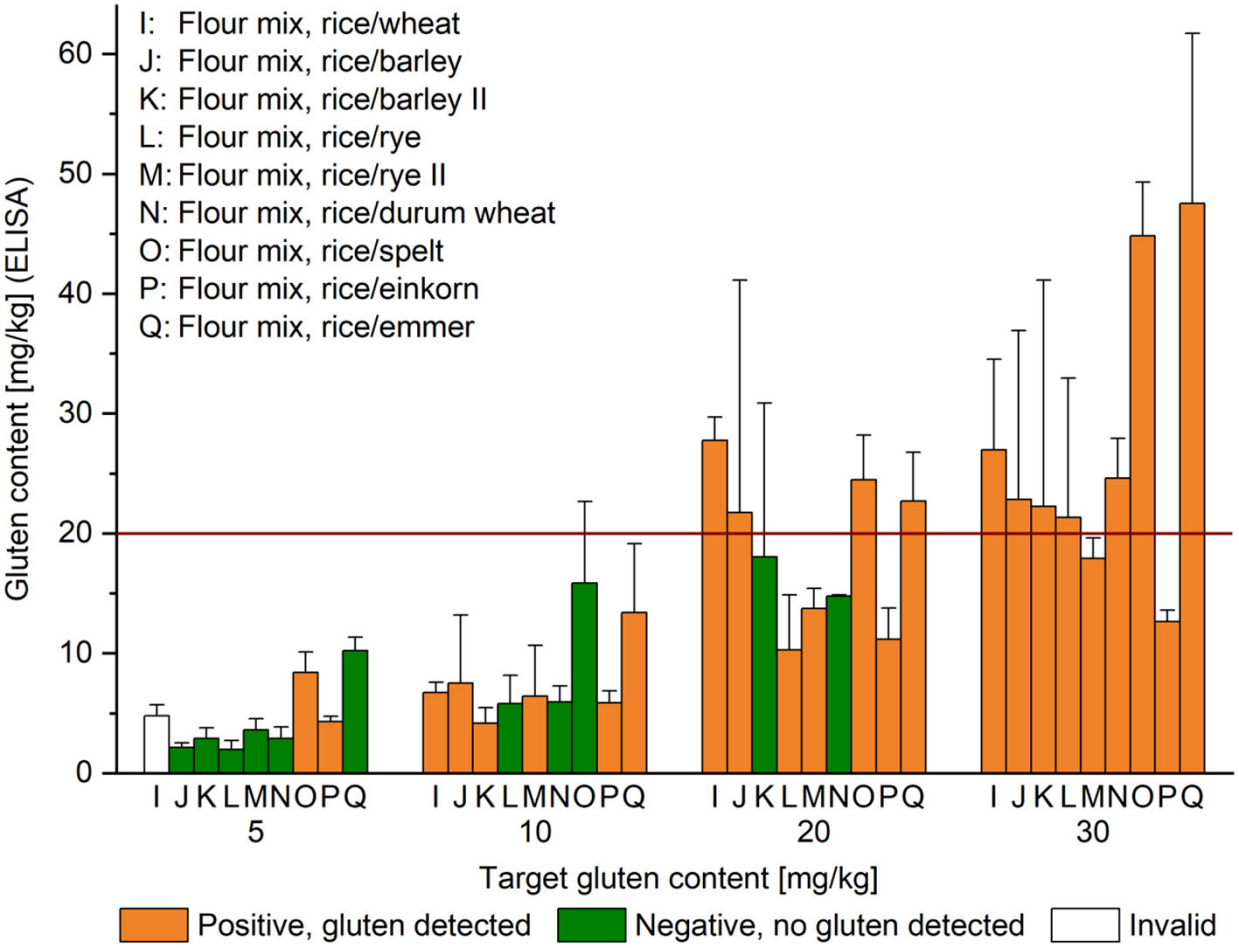
Gluten content of unheated high-starch foods. Target gluten content is indicated below the x-axis and gluten content analyzed by R5 sandwich ELISA on the y-axis; given as mean (*n* = 3) + standard deviation. The result of the sensor at the first of three consecutive measurements was either gluten detected (orange), no gluten detected (green) or invalid (white). Flour mixes designated with (II) were additionally homogenized. The red horizontal line indicates the threshold for gluten-free products at 20 mg/kg.

GF rice flour was mixed by shaking upside down for 12 h with the appropriate amount of wheat, rye, barley, spelt, durum wheat, emmer and einkorn flours as described in Schopf and Scherf (15) (samples I–Q, Figure 2).

#### Heated High-Starch Foods

**Figure 3:**
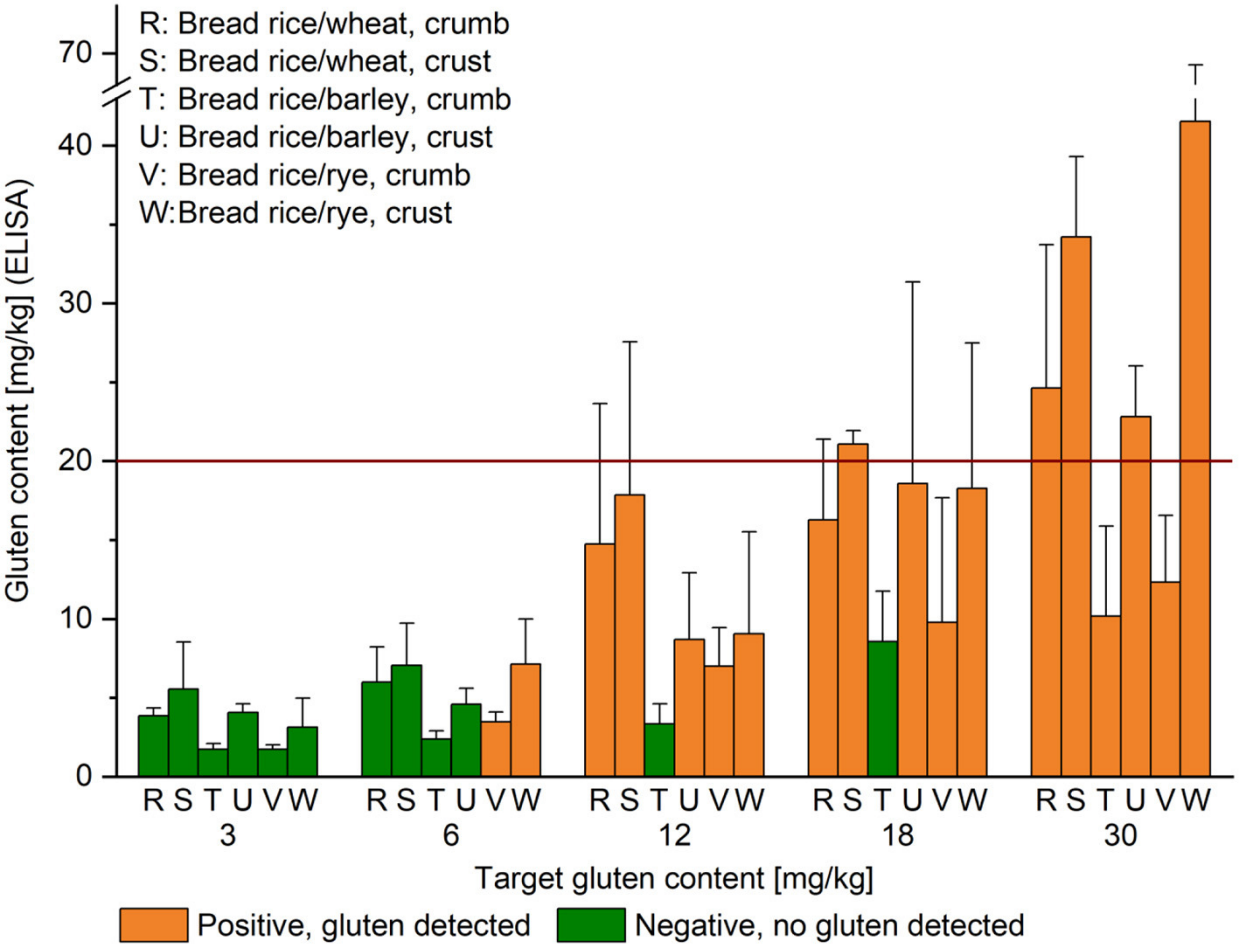
Gluten content of heated high-starch foods. Target gluten content is indicated below the x-axis and gluten content analyzed by R5 sandwich ELISA on the y-axis; given as mean (*n* = 3) + standard deviation. The result of the sensor at the first of three consecutive measurements was either gluten detected (orange) or no gluten detected (green). The red horizontal line indicates the threshold for gluten-free products at 20 mg/kg.

Breads were made from a GF flour mix (Dr. Schär, Burgstall/Postal, Italy), water, dry yeast (Frießinger Mühle, Bad Wimpfen, Germany), sunflower oil and salt. All ingredients were kneaded to a homogeneous dough for 5 min at medium speed using a kitchen machine (MUM4405, Bosch, Munich, Germany). The dough was divided into 150 g portions and either no flour or wheat, rye or barley flour was added followed by further mixing. Then, breads were baked for 35 min at 180°C, removed from the oven, cooled, separated into crumb and crust and cut into small pieces. The pieces were freeze-dried and subsequently homogenized to a fine powder using the blender (crumb samples R, T and V; crust samples S, U and W, Figure 3).

### Gluten Analysis Using ELISA

**Figure 4:**
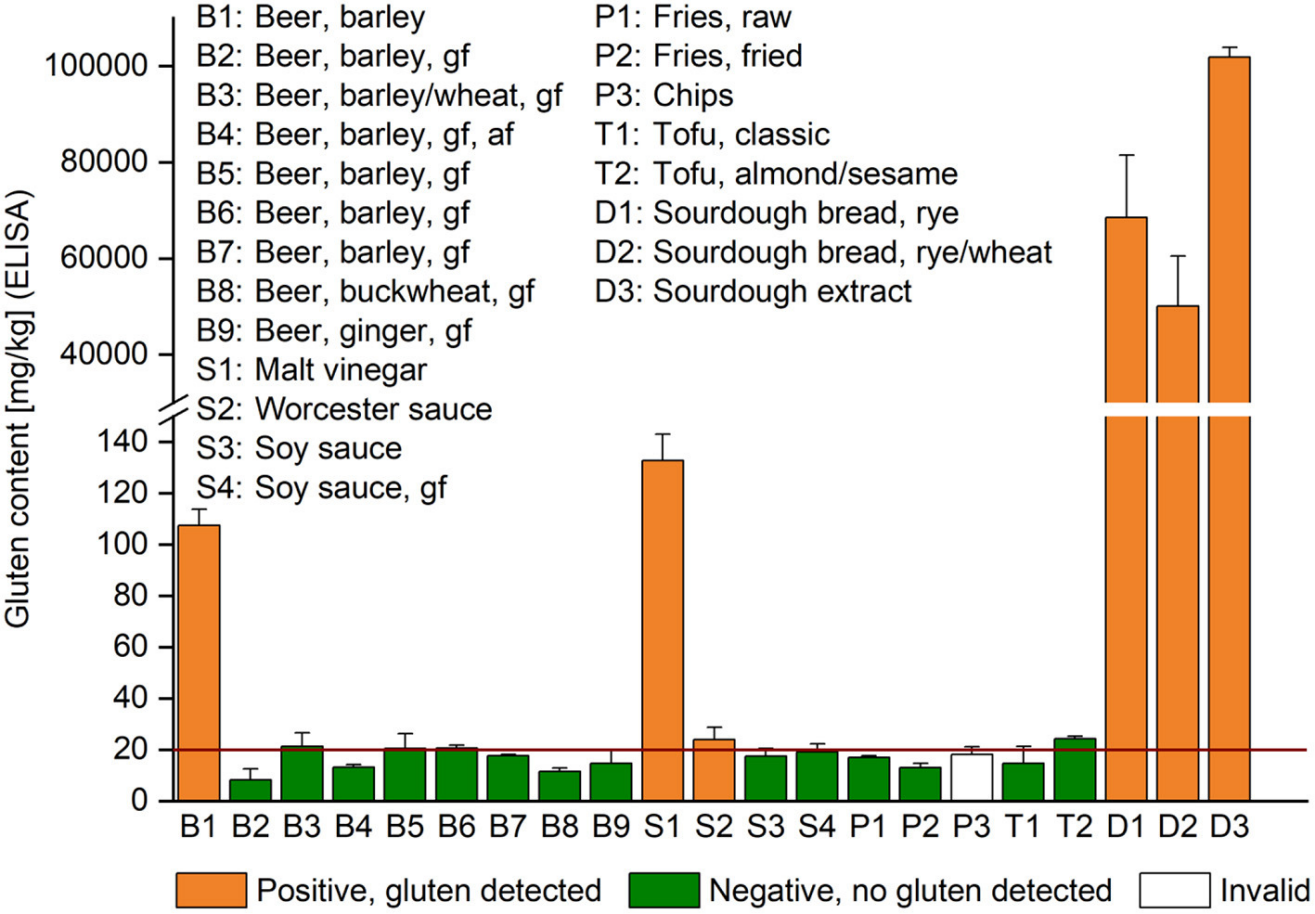
Gluten content of commercially available products with unknown gluten concentrations. The content analyzed by R5 competitive ELISA is given as mean (*n* = 3) + standard deviation. The result of the sensor at the first of three consecutive measurements was either gluten detected (orange), no gluten detected (green) or invalid (white). af, alcohol-free, gf, product with a GF label. The red horizontal line indicates the threshold for gluten-free products at 20 mg/kg.

For comparison, all samples were also analyzed by enzyme-linked immunosorbent assay (ELISA) as reference method. All ELISA measurements were performed in a separate fume hood to avoid gluten contamination and surfaces, vials and equipment had been cleaned with 60% ethanol. The gluten content was determined with three replicates by R5 sandwich ELISA (RIDASCREEN Gliadin; R-Biopharm) for samples A-W (Figures 1–3) or R5 competitive ELISA (RIDASCREEN Gliadin competitive; R-Biopharm) for commercially available products with unknown gluten concentrations (Figure 4). The ELISA was performed strictly according to the manufacturer's instructions, respectively. The absorbances were read at 450 nm with a Tecan Infinite 200 PRO microplate reader (Crailsheim, Germany). The cubic spline function implemented in the software RIDASOFT Win.NET (R-Biopharm) was used to calculate the prolamin content in the samples. Values below the limit of quantitation (2.5 mg/kg for prolamin content) were extrapolated using a second order polynomial function. Gluten content was obtained by multiplying the prolamin content by a factor of 2, as stated in the Codex (3). Homogeneity of selected samples (meatball, wheat, at 20 mg/kg of gluten) prepared to be homogeneous and inhomogeneous, respectively, was tested using ten replicates from different parts of the sample container according to standard procedures (16). Mean values, absolute standard deviations and relative standard deviations (RSD) were calculated for all quantitative results.

### Gluten Analysis Using the Sensor

All food samples were measured in three replicates using the sensor (Nima Labs Inc., San Francisco, CA, USA) strictly according to the manufacturer's instructions. In case of ambiguous results (one replicate not in agreement) three more replicates were analyzed. Sample quantity (0.1–2.0 g, in 0.2 g steps) was varied using four exemplary samples C, H, T, and U (21.3, 15.9, 8.6, and 18.6 mg/kg of gluten, respectively, according to ELISA). To assess whether a high-dose hook effect might occur, wheat, rye, and barley flours were tested directly, as well as the mix_1,000_ of samples B, H, T, T prior to freeze-drying, U, and U prior to freeze-drying. Four different users tested four more exemplary samples F, R, U, and W (5.6, 14.8, 22.8, and 9.0 mg/kg of gluten, respectively, according to ELISA).

## Results

### Gluten Content in Wheat, Rye, and Barley Flours

The wheat flour contained 3.5% gliadins and 3.3% glutenins, amounting to 6.8% gluten (all values based on flour weight). The gluten content of the rye flour was 2.8%, consisting of 1.9% prolamins and 0.9% glutelins, whereas barley had 3.3% of gluten, composed of 0.8% prolamins and 2.5% glutelins. The second sieving step for rye and barley flours (II) resulted in slight changes of total gluten content, so that the rye flour (II) contained 2.5% of gluten and the barley flour (II) 3.9% of gluten. The gluten content of the flours was also analyzed by R5 sandwich ELISA and the results were 7.6% for wheat, 12.0% for rye and 5.6% for barley. This corresponds to recoveries of 112% for wheat, 429% for rye and 169% for barley. The ELISA results of the mix_1,000_ samples were used for further calculations of the final target gluten concentrations.

### Analysis of High-Protein and High-Fat Foods With Defined Gluten Content

Compared to the target gluten content of 4 or 5 mg/kg, 10, 20, and 30 mg/kg for high-protein and high-fat foods, the ELISA results yielded recoveries from 82% (B, meatball, wheat, at 30 mg/kg) to 139% (F, meatball, wheat/rye/barley, at 4 mg/kg) for homogeneous foods. Considering the foods that were intentionally mixed for shorter times to achieve an inhomogeneous gluten distribution, the recoveries were between 91% (C, meatball, wheat, ih, at 10 mg/kg) and 212% (A, sausage meat, wheat, ih, at 5 mg/kg). According to expectations, high RSD of up to 64% in sample H (salad dressing, wheat, ih, at 5 mg/kg) of triplicate determinations were observed for inhomogeneous samples A, C, G, and H (Figure 1). In contrast, the RSD were between 2 and 18% over all homogeneous samples. The sensor returned a result of gluten detected for all samples irrespective of the gluten content at the first of three consecutive measurements. Considering the triplicate measurements with the sensor, there were only 3 negative results out of 96 tests in total (3%). These occurred in samples A at 10 mg/kg, D at 10 mg/kg and F at 5 mg/kg and thus were within the range of measurement uncertainty of the sensor.

### Analysis of Unheated High-Starch Foods With Defined Gluten Content

The comparison of target gluten content and that measured by ELISA resulted in recoveries from 39% (L, rice/rye, at 5 mg/kg) to 204% (Q, rice/emmer, at 5 mg/kg). Additional sieving helped increase recovery to 72% in the rice/rye mix (M, rice/rye II, at 5 mg/kg). The ELISA gave consistently lower recoveries for rice/durum wheat (N, 58–82%) and rice/einkorn (P, 42–86%) mixtures compared to rice/spelt (O, 122–168%) and rice/emmer (Q, 114–204%). All high-starch foods were prepared with the intention to achieve homogeneity, but most RSD lay between 10 and 33%. However, RSD up to 89% (J, rice/barley, at 20 mg/kg) were observed, most likely due to different mixing behavior of the dry powders (Figure 2). The sensor detected gluten in all samples with a target gluten content of 30 mg/kg and in 7 out of 9 samples with 20 mg/kg. One reason may have been inhomogeneity of sample K (rice/barley II), but this explanation does not apply to sample N (rice/durum wheat), because sample N had an exceptionally low RSD (0.5%) at 20 mg/kg. No gluten was detected in 3 out of 9 samples at 10 mg/kg in the samples containing rye (L), durum wheat (N) and spelt (O). At the 5 mg/kg level, the sensor returned the following results: 1 invalid, 2 gluten detected and 6 no gluten found. Out of the 108 triplicate tests with the sensor, there were 74 positive (68.5%) and 33 negative (30.5%) results, as well as 1 invalid result. Only 3 samples (J, L, and N, at 5 mg/kg) always showed a negative result, whereas either 1 or 2 out of 3 tests came back negative for the other samples with a gluten content from 5 to 20 mg/kg. At the threshold of 20 mg/kg, 2 out of 3 tests were negative for sample K and 1 out of 3 for sample N.

### Analysis of Heated High-Starch Foods With Defined Gluten Content

Gluten recoveries assessed by ELISA lay between 28% (T, rice/barley, crumb, at 12 mg/kg) and 185% (S, rice/wheat, crust, at 3 mg/kg). As already reported for the unheated high-starch foods, some heated samples also had high RSD with up to 81% (V, rice/rye, crumb, at 18 mg/kg), but others as low as 4%, with most between 10 and 38%. The gluten sensor found gluten in almost all samples with a target gluten content of 12 mg/kg or higher, except for sample T at 12 and at 18 mg/kg (Figure 3). No gluten was detected in any of the samples at the 3 mg/kg level. This was according to expectations for samples T and V that also tested below 2 mg/kg by ELISA. Gluten detection might have been possible for the other samples at this level, because the gluten content analyzed by ELISA was 3.1 mg/kg or higher, but the sensor returned only negative results also after triplicate analysis. Two out of 6 samples (V and W) tested positive at the 6 mg/kg level at the first of three measurements and in 5 out of 6 tests in total. Of the samples that tested negative, the sensor found no gluten in 3 out of 3 replicates in samples T and U, whereas it found no gluten in 2 out of 3 replicates in sample R and in 1 out of 3 replicates in sample S. Overall, of the 90 tests performed, 55 (61%) came back as gluten found, 34 (38%) as no gluten detected and 1 as invalid. As observed before, the sensor detected gluten also well below 20 mg/kg.

### Analysis of Foods With Unknown Gluten Content

A selection of commercially available foods with unknown gluten content was also tested with the sensor to study whether it could also detect fermented and partially hydrolyzed gluten. The gluten content was analyzed by competitive ELISA for comparison (Figure 4). One regular barley-based beer (B1) had a gluten content of 107 mg/kg and it tested positive using the sensor. All other barley-based beers (B2-B7) had a GF label according to European legislation and they tested negative using the sensor. The ELISA values were between 8.2 mg/kg (B2) and 21.3 mg/kg (B3). Two beers from naturally GF raw materials were also included (B8, B9), but to our surprise, the ELISA still detected 11.6 and 14.7 mg/kg of gluten, whereas the sensor did not. Among the sauces, both sauces with gluten concentrations above 20 mg/kg (S1, S2) tested positive using the sensor, whereas the other two with 17.5 mg/kg (S3) and 19.1 mg/kg of gluten (S4) did not. All potato and tofu samples returned a negative or invalid result using the sensor, while the ELISA detected between 13.1 mg/kg (P2) and 24.2 mg/kg (T2) of gluten. None of these samples had a GF label. All three sourdough samples had extremely high gluten concentrations of 50,097 mg/kg or higher and they were clearly identified as gluten-containing samples using the sensor.

### Influence of Sample Weight and High Gluten Content on the Results of the Sensor

There was no evidence for a dependence of the results on the sample weight from 0.1 to 1.5 g for samples C and H, because the sensor detected gluten in all cases. For sample C, even higher sample weights of up to 2.0 g were possible and the intensity of the test line relative to that of the control line increased with increasing sample weight. In case of sample H, 1.5 g was the maximum, because otherwise the viscosity became too high. Starch-rich foods T and U had a smaller working range from 0.3 to 0.9 g, because the capsules could not be closed anymore with higher amounts and the result using only 0.1 g came back negative.

**Figure 5:**
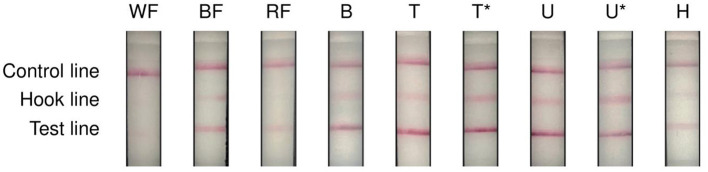
Results of the sensor when testing high gluten concentrations. WF, wheat flour with 6.8% of gluten, BF, barley flour with 3.3% of gluten, RF, rye flour with 2.8% of gluten, B, meatball wheat, T, bread rice/barley, crumb, T*, bread rice/barley, crumb prior to freeze-drying, U, bread rice/barley, crust, U*, bread rice/barley, crust prior to freeze-drying, H, salad dressing, wheat, inhomogeneous. Samples B, T, T*, U, U* and H contained 1,000 mg/kg of gluten.

The sensor detected gluten in all analyses of samples containing 1,000 mg/kg or even higher, as in the wheat, rye, and barley flours (Figure 5). However, while the intensity of the control line was mostly comparable, the intensity of the hook line was comparatively weak. The test line appeared intensely in all samples containing 1,000 mg/kg and it did not appear to make a difference whether the sample had been freeze-dried or not. When wheat or rye flours were tested directly, the test line was barely discernible, whereas barley flour seemed to be detected more clearly compared to wheat and rye.

### Influence of Different Users on the Results of the Sensor

**Figure 6:**
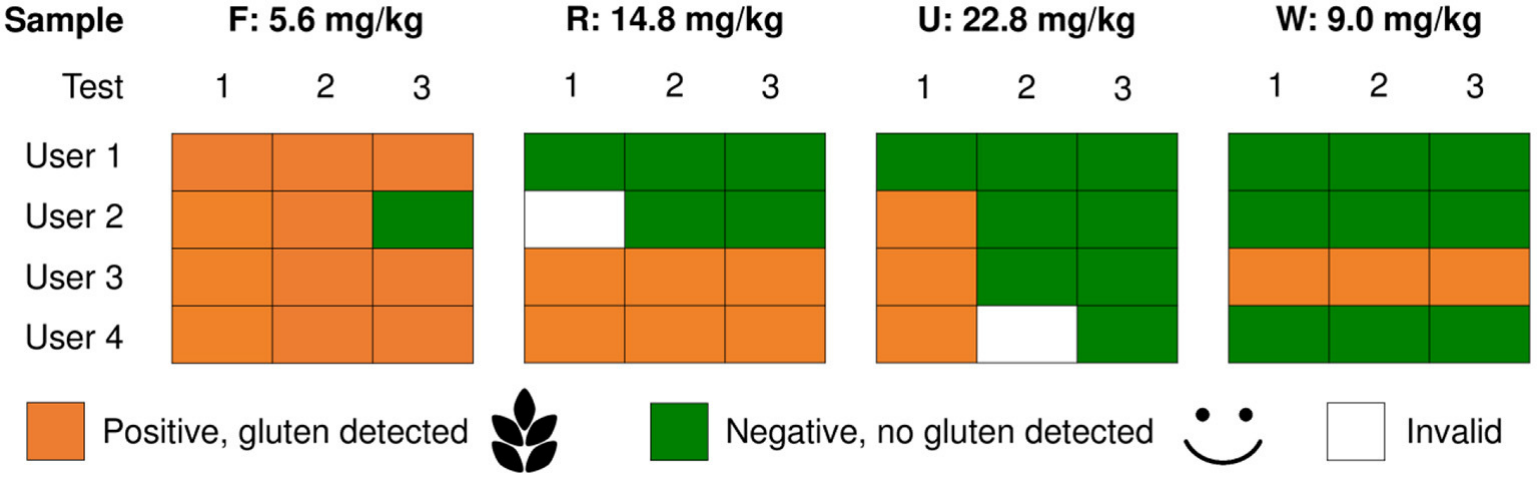
Results of the sensor depending on the user. Samples F (meatball, wheat/rye/barley), R (bread rice/wheat, crumb), U (bread rice/barley, crust) and W (bread rice/rye, crust) were tested three times (test 1, 2 and 3) each by four different users.

The results of the sensor showed systematic variability between different users that also appeared to depend on the sample (Figure 6). While all four users detected gluten in sample F in 11 out of 12 measurements, only one user consistently detected gluten in sample W, whereas all others did not. The results were even less reliable for samples R and U, because two users detected gluten in sample R, whereas two did not. For sample U, the results indicated that three out of four users detected gluten using the sensor in <33% of cases.

## Discussion

Of the 119 samples with gluten content ranging from 2 to 101,888 mg/kg tested in total, the sensor showed 80 positive (67.2%), 37 negative (31.1%) and 2 invalid results at the first of three consecutive measurements. When considering all three replicates amounting to 357 tests in total, the percentages remained similar, because there were 241 positive (67.5%), 113 negative (31.7%) and 3 invalid results. Therefore, we decided to focus on the first measurement, because users are unlikely to analyze the same food more than once due to time and cost limitations. About 50% of adults and 86% of teenagers agreed that the test was time-consuming (17) and some commented that the price per capsule was too high (18).

Our detection rate of 90% for samples containing ≥20 mg/kg was comparable to the 87.5% reported by Taylor et al. (11) but somewhat lower than the 99% (confidence interval 97.8–100%) claimed by the manufacturer (10). The sensor should report a GF result for samples containing <2 mg/kg and this was also the case in our study for samples R and T and for the GF raw materials (results not shown). However, samples with up to 18.0 mg/kg (K, intact gluten in the rice/barley flour mix) and 24.2 mg/kg (T2, most likely with partially hydrolyzed gluten) also returned a GF result. In case of sample K, this is deemed acceptable, because the gluten content was still below the regulatory threshold of 20 mg/kg, but not for sample T2. Regarding different sources of gluten, the sensor detected gluten from all species tested, but it appeared to be less sensitive to durum wheat (sample N).

Due to its sandwich design using two antibodies, the manufacturer acknowledges that the sensor may incorrectly show a negative result when fermented foods such as beer, soy sauce and malt extracts/flavorings are tested. Despite this, the test reported gluten in barley-based beer, malt vinegar, Worcester sauce and sourdough extracts. From the samples tested, it appeared that the sensor did detect partially hydrolyzed gluten in foods, but with lower sensitivity compared to intact gluten. This issue needs to be communicated very clearly to the users, because it is not always easy for them to determine whether a composite food containing gluten of unknown origin may contain partially hydrolyzed, fermented or fractionated gluten and may thus cause false-negative results. However, when users where asked to recall the device's testing limitations, nearly half of those asked could not correctly identify these limitations (17). This deficit in user knowledge and education needs to be addressed adequately to help prevent giving a false sense of security. A recent systematic review identified increased patient education/physician-patient communication and increased knowledge of a GF diet as the two most significant facilitators contributing to improved adherence to a GF diet, while lower knowledge of CD and restaurant dining/supermarket shopping were the two most significant barriers (19).

The result of “gluten found” in 56% of samples with <20 mg/kg was according to expectations (10, 11), but it is still likely to cause confusion and also unnecessary anxiety among users, because even samples with a GF label may test positive using the sensor. As trust in the results of the sensor was generally high, ranging from 77 to 100% in adults and teenagers, respectively, over 65% of users reported that the sensor indicated “gluten found” for foods that they had thought to be GF (17). Consequently, they did not eat these foods and might therefore limit an already restrictive diet even more. Developing a qualitative test with high diagnostic accuracy that classifies samples with a gluten content below 20 mg/kg as GF and those above as gluten-containing is certainly demanding. However, this should be encouraged for further improvements, because the result finally leads CD patients in their decision making whether to consume the food or not.

Using a gluten sensor may affect individual CD patients in different ways. More than 90% of both adults and teenagers agreed that it helped them follow a GF diet and gave peace of mind. CD quality of life (QOL) scores improved for adults, but remained unchanged for teenagers. In contrast, 43% of teenagers reported that using the sensor made them anxious (17). Future studies could be designed in a way to evaluate if using a gluten sensor contributes to more accurate gluten avoidance by CD patients compared to those that do not have access to any portable device. The connection between user experience with the sensor, CD QOL and long-term mucosal healing needs to be investigated further, as also suggested by Wolf et al. (18), especially in light of the ongoing debate of how strict a GF diet needs to be.

On the one hand, recent findings indicate that occasional and voluntary low level gluten consumption was not associated with the onset of CD symptoms, serology or histology in a group of asymptomatic adult CD patients (20). Moreover, strict compliance to a GF diet has been reported to decrease QOL compared to the general population and may be low in patients, especially during social events (21). On the other hand, the CD QOL score tended to be higher in patients adhering to the GF diet compared to non-compliant subjects (22). Therefore, the benefits and potential risks of using a portable gluten sensor need to be carefully evaluated and weighed to provide tailored individual recommendations to help CD patients manage their GF diet in the least restrictive way possible.

Acknowledged limitations of our study include a focus on protein- and starch-rich foods, small sample size and subsampling of foods, some of which had inhomogeneous gluten distribution. Further, all users were non-CD patients and they knew of their study participation. This introduces a bias toward very careful use of the sensor in an analytical laboratory setting as opposed to a real life setting. Therefore, the performance of the sensor is likely to be more reliable in our well-controlled study conditions compared to daily routine use, e.g., in a restaurant or a canteen.

Overall, the performance of the sensor was acceptable in our study, but the systematic variation observed between different users was concerning. This could be related to difficulties with inserting a food sample of appropriate size or difficulty in closing the capsules without using the wrench, as has been reported by users (18). Further testing of the same samples with a higher number of different users would also be helpful to identify systematic factors affecting the results obtained with the sensor and improve instructions for use.

For some samples, repetitive testing gave inconsistent results, most likely due to inhomogeneous distribution of gluten in the sample. Correct sampling directly affects testing reliability, but it is difficult to issue clear guidance for composite foods, such as those present in a real life restaurant setting. This is a general point that limits the applicability of handheld or smartphone-based devices in the hands of CD or food allergy patients.

In conclusion, the gluten sensor may be useful for CD patients to test foods for peace of mind, especially when eating out. The handheld device comes with a charging cable and is easy to carry during travel. However, user education is of critical importance and has to be improved, because users need to be aware of testing limitations, such as the effect of sampling and the potential occurrence of partially hydrolyzed, fractionated or fermented gluten.

## Data Availability Statement

The original contributions presented in the study are included in the article/supplementary material, further inquiries can be directed to the corresponding author/s.

## Author Contributions

AM: formal analysis, investigation, data curation, and writing—review and editing. KS: conceptualization, funding acquisition, resources, supervision, and writing—original draft. All authors reviewed and approved the final manuscript.

## Conflict of Interest

The authors declare that the research was conducted in the absence of any commercial or financial relationships that could be construed as a potential conflict of interest.
